# Prognostic Significance of Lymph Node Ratio in Intrahepatic and Extrahepatic Cholangiocarcinomas †

**DOI:** 10.3390/cancers17020220

**Published:** 2025-01-11

**Authors:** Andrii Khomiak, Sumaya Abdul Ghaffar, Salvador Rodriguez Franco, Ioannis Asterios Ziogas, Ethan Cumbler, Ana Luiza Gleisner, Marco Del Chiaro, Richard David Schulick, Benedetto Mungo

**Affiliations:** Division of Surgical Oncology, Department of Surgery, University of Colorado School of Medicine, Aurora, CO 80045, USA; andrii.khomiak@cuanschutz.edu (A.K.);

**Keywords:** lymph node ratio, intrahepatic cholangiocarcinoma, extrahepatic cholangiocarcinoma, survival, prognosis, lymphadenectomy

## Abstract

*Background/Objectives*. Lymph Node Ratio (LNR) is increasingly recognized as an important prognostic factor in various cancer types, with the potential to enhance patient stratification for intrahepatic (ICC) and extrahepatic (ECC) cholangiocarcinoma. The study aimed to assess the impact of LNR on survival in surgically resected patients with ICC and ECC. *Methods*. This was a retrospective analysis of National Cancer Database (2004–2020) included ICC and ECC (excluding distal bile duct) patients who underwent primary site resection with adequate lymphadenectomy (≥4 LNs excised). Exclusions comprised age < 18 years, distant metastasis, or incomplete key data. LNR was calculated as the ratio of positive LNs to total examined LNs. Survival probabilities were estimated using Kaplan–Meier analysis and Cox regression. *Results*. The inclusion criteria were met by 954 patients with ICC and 1607 patients with ECC. In patients with ICC, the median OS time was 62.7 months in LNR0 group, 40.8 months in LNR < 30%, and 25.2 months in LNR ≥ 30% (*p* < 0.001). In ICC, 3-year OS was 69.3%, 54.6%, and 34% for LNR 0, LNR < 30%, and LNR ≥ 30%, respectively (*p* < 0.05). When adjusted for age, sex, Charlson–Deyo score, histology, surgical margins, chemo- and radiotherapy using Cox regression, LNR < 30% and LNR ≥ 30% were associated with worse OS in patients with ICC (HR 2.1 (95% CI 1.6–2.7) and HR 2.94 (95% CI 2.3–3.8)) and ECC (HR 2.1 (95% CI 1.8–2.5) and HR 3 (95% CI 2.4–3.7)). *Conclusions*. It is well-known that LN-negative patients have significantly better survival than LN-positive patients with ICC and ECC. This study strongly demonstrates that survival prognosis can be further stratified based on LNR for ICC and ECC patients and that it is not simply a binary factor.

## Simple Summary

Cholangiocarcinoma is a rare cancer affecting the bile ducts in the liver. While it is known that cancer spreading to lymph nodes often leads to a worse prognosis, simply determining whether lymph nodes are positive or negative may not provide the full picture. This study investigated whether the proportion of affected lymph nodes—known as the lymph node ratio—can better predict patient survival after surgery in patients with intrahepatic and extrahepatic cholangiocarcinoma. Findings suggest that a higher lymph node ratio correlates with decreased overall survival, highlighting its potential role in guiding treatment strategies.

## 1. Introduction

Cholangiocarcinoma is a rare cancer that originates from the biliary tree, predominantly extrahepatically (ECC), with intrahepatic (ICC) occurrence observed in less than 10% of cases [1]. While only 20–30% of patients present with potentially resectable tumors, survival rates remain dismal even after surgery, with only approximately 39% 5-year survival for ICC and up to 47% for ECC [1,2,3,4]. One of the most important prognostic factors for resectable cholangiocarcinomas is the presence of lymph node metastasis [5]. The most recent edition of the American Joint Committee on Cancer (AJCC) altered the nodal staging for ECC, transitioning from the binary presence or absence of metastasis to a stratification based on the number of compromised nodes [6,7]. However, the N staging for ICC remains unchanged, considering any positive nodal involvement as N1 disease [8].

Despite its relevance, the AJCC staging system has its pitfalls [9,10,11], In fact, this focuses solely on the number of compromised nodes, while not accounting for the total number of lymph nodes removed (which can vary widely) and the extent of disease spread within examined lymph nodes [10], For instance, one node positive out of one removed may hold a different prognostic value than one node positive out of ten removed. To address this limitation, the assessment of the lymph node ratio (LNR) and the log odds of positive lymph nodes (LODDS) was initially applied to other cancers, resulting in improved survival predictions [12,13,14]. The role of LNR has been explored in cholangiocarcinoma through studies with small samples. Among others, Oshiro et al. evaluated 60 patients with extrahepatic cholangiocarcinoma and found that higher LNR was an independent predictor of survival, with five-year survival rates of 44% for patients with an LNR of 0, dropping to 10% for LNRs of 0–0.2, and 0% for LNRs ≥ 0.2. In contrast, Sakata et al. analyzed 59 patients and showed that the number of positive lymph nodes, rather than LNR, was a superior prognostic factor in adequately staged patients. [15,16,17] While studies like Sakata et al. highlight limitations in specific contexts, the broader body of evidence supports the prognostic utility of LNR, underscoring the need for large, comprehensive studies to clarify its role in cholangiocarcinoma.

Detailed nodal staging is crucial not only for prognosis evaluation but also for defining subsequent adjuvant therapy choices [18,19]. Incorporating tools such as LNR into cholangiocarcinoma staging systems could enhance their accuracy. This study aims to assess the impact of LNR on the survival of patients undergoing intrahepatic and extrahepatic cholangiocarcinoma resection at a national level, exploring the feasibility of the approach for nodal staging.

## 2. Methods

### 2.1. Study Design and Population

The study utilized data from the National Cancer Database (NCDB), the result of collaborative efforts between the Commission of Cancer of the American College of Surgeons and the American Cancer Society. It comprises oncology outcomes from over 1500 CoC-accredited cancer programs in the United States, encompassing more than 70% of all newly diagnosed cancer cases in the country. The database data were used for the period spanning 2004 to 2020. International Classification of Diseases, 10th revision, codes were used to identify intrahepatic bile duct carcinoma (C22.1) and malignant neoplasm of the extrahepatic bile duct (C24.0). Distal bile duct cancers were excluded from the analysis using site-specific code due to variations in the lymphadenectomy procedure for this disease, particularly in the number of nodes considered for minimal accurate staging [7]. Histologic codes included were 8140 (adenocarcinoma, NOS), 8160 (cholangiocarcinoma) and 8162 (Klatskin tumor). We only included patients with curative intent procedures of the primary site (resection).

We excluded patients < 18 years old, patients with metastatic disease and missing or incomplete data on staging, procedure of primary site, extent of lymphadenectomy, chemotherapy, and survival. The extent of adequate lymphadenectomy for both ICC and ECC is a point of debate, with no common thresholds adopted for a minimum number of LNs resected. For ICC, suggested cutoffs range from 3 to 6 LNs, while for the ECC, cutoffs from 4 to 7 LNs are proposed [7,8]. For our study, the cutoff of 4 or more examined LNs was selected, and patients with less than 4 LNs examined were excluded from analyses.

### 2.2. Variables and Definitions

Analyzed variables included demographics, facility type, distance traveled, and Charlson–Deyo Score. Cancer-related variables included histology, AJCC TNM staging [20], the extent of surgery/lymphadenectomy, number of lymph nodes removed and examined, surgical margins, chemotherapy status, and radiotherapy receipt. Lymph node ratio was defined as the number of positive lymph nodes divided by the total number of examined lymph nodes. A cutoff value of 0.3 (30%) for LNR for both ICC and ECC was chosen based on previous reports [15,21,22,23,24] and results of preliminary analyses indicating the best discrimination within our cohort. Patients were divided into three groups based on LNR: LNR 0, LN < 30% (lower risk), and LN ≥ 30% (higher risk). The value of log odds of disease spread (LODDS) was explored as a supplementary measure of lymph node involvement. LODDS was determined as the logarithm of the ratio between the number of positive nodes and the number of negative nodes and calculated using the formula log[(positive LNs + 0.5)/(total LNs–positive LNs + 0.5)]; 0.5 is added to both the numerator and denominator to avoid division by zero in cases with no positive lymph nodes[25]. Patients were categorized into the following LODDS groups: LODDS0 (LODDS ≤ −2.3) was the group with the lowest risk; LODDS1 (−2.3 < LODDS ≤ −0.6) constituted an intermediate risk group; and LODDS2 (LODDS > −0.6) identified patients with the highest risk.

### 2.3. Statistical Analysis

Continuous variables were expressed using medians with interquartile ranges or means with standard deviations. The Kruskal–Wallis test was used to compare continuous variables. Chi-square was used to compare categorical variables. Overall survival (OS) was calculated based on the time from the date of diagnosis to the date of death or last contact (censored). Kaplan–Meier survival analysis with the log-rank test was used to compare the survival outcomes between different LNR and LODDS groups. Cox regression survival analysis was utilized to adjust for potential confounders and identify risk factors for OS. A *p*-value of less than 0.05 was considered significant for all tests. Given that the study data consists of de-identified information from NCDB, it was considered exempt from Institutional Board Review. All analyses were performed using IBM SPSS Statistics version 29.

## 3. Results

### 3.1. Patient Demographics and Baseline Characteristics

**Figure 1:**
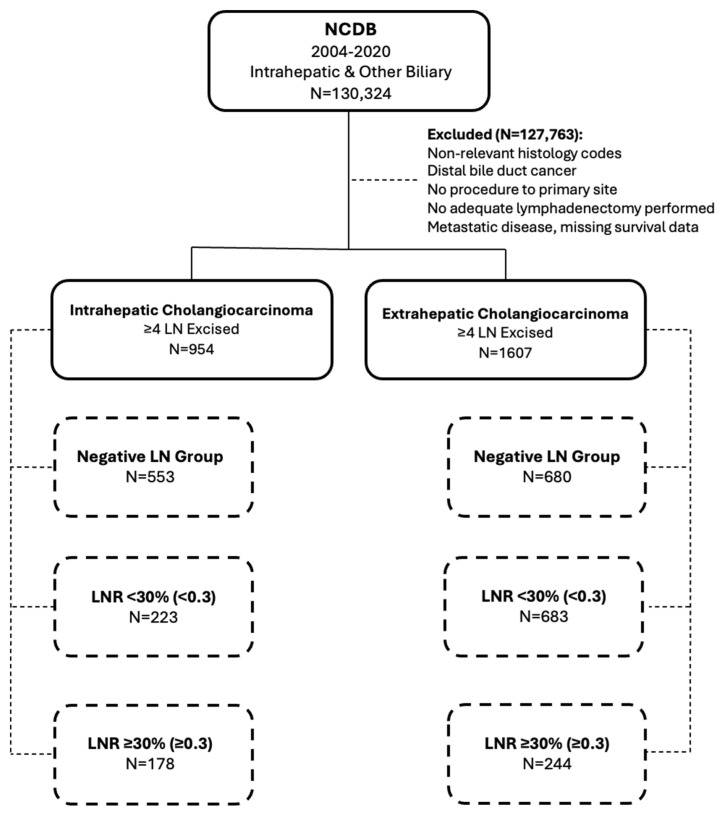
Study Flowchart.

The study included 954 patients with ICC and 1607 patients with ECC (Figure 1). Demographics and baseline characteristics by LNR group are described in detail in Table 1. Patients with ICC and an LNR ≥ 30% were generally younger, with a median age of 63 years (55–70 IQR), with a lower proportion of patients aged 65 or older (79 patients, 44.4%) compared to those with negative lymph nodes, who had a median age of 66 years (58–73 IQR, *p* = 0.02), and 305 patients (55.2%, *p* = 0.03) aged ≥ 65. There were no other significant differences in age, sex, race, facility type, or distance traveled in patients with ICC and ECC across LNR groups. The ICC LNR < 30% group had fewer comorbidities, with 166 patients (74.4%) having a Charlson–Deyo Score of 0, compared to 122 patients (68.5%) in LNR ≥ 30% group and 369 patients (66.7%) in the LNR 0 group (*p* = 0.03). The comorbidity index did not differ significantly across LNR groups in ECC patients.

### 3.2. Cancer Staging

A significant portion of data regarding pathological TNM staging is missing in NCDB. From present data, ICC patients in LN-positive groups tended to have more advanced pT3 and pT4 stages—34.6% in LNR < 30% and 33.8% in LNR ≥ 30% vs. 17.4% in LNR 0 (*p* < 0.001). A similar trend was seen in patients with ECC: 53.8% of patients in LNR < 30% and 48.4% in LNR ≥ 30% had pT3 or pT4 disease, with only 21.3% of either of these stages diagnosed in patients with LNR 0s (*p* < 0.001). In the ICC, an average of 7.8 LNs were examined in the LNR 0 group, 12 LNs in LN < 30%, and 8.1 in LNR ≥ 30% (*p* < 0.001). Similar distribution was noted in ECC patients, with 14.9 LNs on average examined in LNR 0s, 19.2 LNs in LNR < 30%, and 12.5 LNs in LNR ≥ 30% (*p* < 0.001). As expected, patients in higher-risk LNR groups had more positive LNs and thus more advanced pN stages, although for ICC there is no N2 stage to indicate more advanced nodal disease in the current AJCC classification.

### 3.3. Positive Surgical Margins and Adjuvant Treatment

Correlating with more advanced pT stages, patients in the LNR < 30% and ≥30% groups were more likely to have positive surgical margins. In ICC, R1 or R2 resection were performed in 34 patients (15.2%) with LNR < 30% and 43 patients (24.1%) with LNR ≥ 30% compared to 68 patients (12.3%) in patients with no positive nodes (*p* < 0.001). In ECC, positive margins were present in 101 patients (14.7%) with LNR < 30% and 63 (25.8%) patients with LNR ≥ 30% vs. 76 patients (11.2%) in LNR 0 group (*p* < 0.001).

Both ICC and ECC lymph node-positive patients were more likely to receive some regimen of chemotherapy. In ICC patients, adjuvant chemotherapy was administered to 209 patients (37.8%) with LNR 0s, 131 patients (58.7%) in LNR < 30%, and 104 patients (58.4%) in LNR ≥ 30% (*p* < 0.001). Adjuvant chemotherapy for ECC was received by 338 patients (49.7%) with LNR 0s, 439 patients (64.3%) with LNR < 30%, and 167 patients (68.4%) with LNR ≥ 30%. Neoadjuvant-only chemotherapy, conversely, was administered to a smaller proportion of patients in higher-risk LNR groups, likely due to the decision to complement it with an adjuvant regimen after discovering positive LNs. Still, a total of 50 (22.4%) and 36 patients (20.2%) with ICC, as well as 186 (27.2%) and 64 patients (26.2%) with ECC, did not receive any chemotherapy in LNR < 30% and LNR ≥ 30% groups, respectively. The majority of patients, 747 (78.3%) with ICC and 1122 (69.8%) with ECC, did not receive any radiation treatment. However, patients with positive LNs were more likely to receive adjuvant radiation for both ICC and ECC.

### 3.4. Survival Analysis

**Figure 2:**
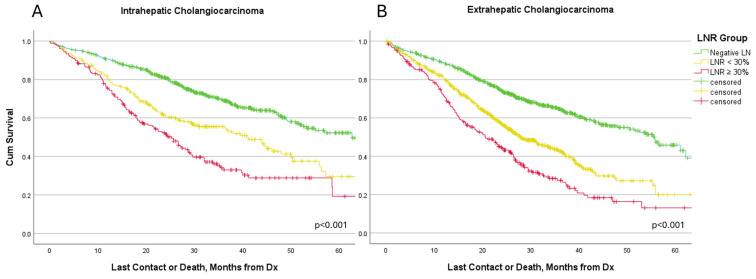
Kaplan–Meier Survival Curves for LNR Groups. (**A**)—Intrahepatic Cholangiocarcinoma, (**B**)—Extrahepatic Cholangiocarcinoma.

In our cohort, LNR was a strong predictor of survival (Figure 2, Table 2). In patients with ICC, the median OS time was 62.7 months in LNR 0 group, 40.8 months in LNR < 30%, and 25.2 months in LNR ≥ 30% (*p* < 0.001). In ICC, 3-year OS was 69.3%, 54.6%, and 34% for LNR 0, LNR < 30%, and LNR ≥ 30%, respectively (*p* < 0.05). Patients with ECC had 55.7 months median OS in LNR 0 group, 27.9 months in LNR < 30%, and 20.5 months in LNR ≥ 30%. The 3-year survival for ECC patients was 64.7% in LNR 0, 42.6% in LNR < 30%, and 27.5% in LNR ≥ 30% (*p* < 0.05). LODDS was also found to be a robust predictor of survival outcomes (Table 2).

**Figure 3:**
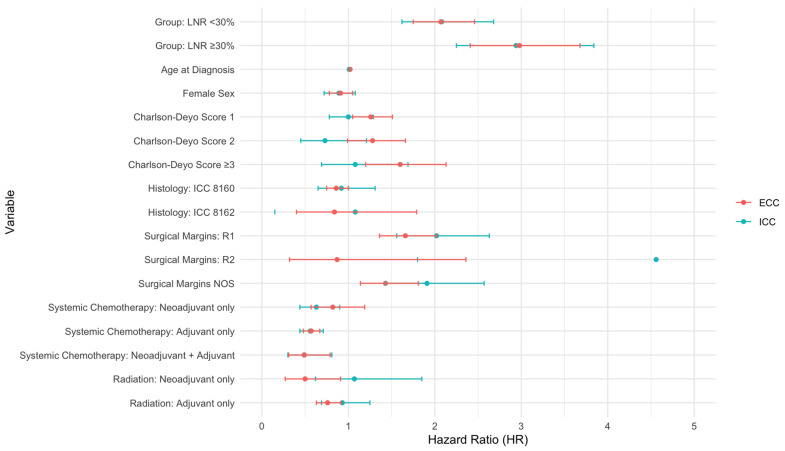
Forest Plot: Hazard Ratios for ICC and ECC.

In Cox regression analysis, LNR was found to be predictive of survival in both univariable and multivariable analyses (Table 3, Figure 3). When adjusted for potential confounders, i.e., age, sex, Charlson–Deyo score, histology, surgical margins, chemotherapy and radiotherapy, LNR < 30% and LNR ≥ 30% were associated with worse OS in patients with ICC (HR 2.1 [95% CI 1.6–2.7] and HR 2.94 [95% CI 2.3–3.8]) and ECC (HR 2.1 [95% CI 1.8–2.5] and HR 3.0 [95% CI 2.4–3.7]). In the same multivariable model with LNR < 30% taken as a reference, LNR ≥ 30% was associated with significantly worse OS (HR 1.53 [95% CI 1.2–2] for ICC and HR 1.48 [95% CI 1.2–1.8] for ECC).

### 3.5. Factors Associated with Survival

Other factors associated with worse OS in multivariable Cox regression were Charlson–Deyo Score for ECC (HR 1.6 [95% CI 1.2–2.1] for score ≥ 3), as well as positive surgical margins in ICC (HR 2.0 [9 5% CI 1.6–2.6] for R1 and HR 4.6 [1.8–11.6] for R2) and ECC (HR 1.66 [95% CI 1.4–2] for R1). In our cohort, systemic chemotherapy was associated with improved OS in multivariable analysis in ICC for neoadjuvant only (HR 0.63 [95%CI 0.44–0.9]), adjuvant only (HR 0.56 [95% CI 0.44–0.71]), and neoadjuvant + adjuvant (HR 0.49 [95% CI 0.3–0.81]) regimens. For ECC, adjuvant chemotherapy (HR 0.57 [95%CI 0.48–0.67]) and neoadjuvant + adjuvant regimen (HR 0.49 [95% CI 0.31–0.79]) also showed a significant association with improved survival in multivariable analysis. Neoadjuvant radiation (HR 0.5 [95% CI 0.27–0.91]) and adjuvant radiation (HR 0.76 [95% CI 0.63–0.92]) were associated with survival benefit in patients with ECC.

## 4. Discussion

This study represents one of the largest cohorts for both intrahepatic and extrahepatic resected cholangiocarcinomas, allowing an evaluation of outcomes on a population-wide scale. The role of lymph node evaluation has evolved over time and varies across different cancer types. While lymph node status plays a well-established role and has a pronounced impact on prognosis in gastric, colorectal, and pancreatic cancer, studies on cholangiocarcinomas and gallbladder cancer yield conflicting results [26,27,28,29]. The role of lymphadenectomy in the management of cholangiocarcinomas has been the subject of debate, with few studies indicating no additional survival benefit [18,30]. Nevertheless, in clinical practice, the need to perform adequate lymphadenectomy remains widely recognized as a crucial step in accurate staging and prognostication of the disease, leading to its incorporation into guidelines [31].

The optimal number of nodes required for a thorough evaluation of nodal status has also been a matter of debate, with varying numbers proposed in the literature [32,33]. In the latest edition of the AJCC, the staging system underwent a significant transformation, shifting from the prior binary definition, based on the presence or absence of metastatic nodes in the pathological evaluation, to a more nuanced approach that considers the number of compromised nodes [6,7]. The updated system also specifies the optimal number of nodes needed for thorough pathological evaluation, setting the minimum at 6 nodes for ICC and 12 nodes for distal cholangiocarcinoma [7,8]. While the staging system for perihilar disease was altered in the latest edition, AJCC authors did not explicitly designate the optimal number of nodes for this specific scenario [6]. Recommended adequate numbers in the literature vary from 3 to 7 [11,18,34]. In our cohort, we chose only to include patients with at least four nodes examined for both ICC and ECC to test the performance of LNR and LODDS under favorable conditions as both calculations depend on the total number of nodes resected. This choice is particularly relevant given that the adequacy of lymphadenectomy is not consistently achieved, especially in the Western world, where the procedure is still underperformed [35]. According to a recent NCDB-based report, only 17.2% of patients had more than five nodes removed for ICC, and having an adequate number of nodes removed was associated with stronger odds of detecting metastasis in at least one node (OR 2.63 [95% CI 2.25–3.08]) for ICC, irrespective of preoperative nodal status [36].

Patients with ICC and ECC who are LN-negative are known to have significantly better survival outcomes than those with LN-positive disease [34,37]. In recent years, more emphasis has been placed on understanding the relationship between positive nodes and the total number of nodes retrieved, considering that the number of positive nodes serves as an independent prognostic factor for both ICC [38,39,40,41] and ECC [5,42]. Similarly to nodal staging in other cancers, the application of LNR to the evaluation of cholangiocarcinomas followed its utilization in diverse cancer types [12,13,14,43]. In our cohort, LNR groups showed strong discrimination of 3-year OS in ICC—54.6% vs. 34% for LNR < 30% compared to ≥30%, and ECC—42.6% in LNR < 30% vs. 27.5% in LNR ≥ 30% (*p* < 0.05). LNR continued to demonstrate a strong association with survival for ICC (HR 2.94 [95% CI 2.3–3.8] for LNR ≥ 30%) and ECC (HR 3 [95% CI 2.4–3.7] for LNR ≥ 30%) after adjusting for multiple confounders, including adjuvant chemotherapy and surgical margins, with the latter being a well-established factor that compromises survival when R0 resection is not achieved (*p* < 0.001) [44,45]. Notably, for hepatobiliary cancers, LNR demonstrated superior performance in predicting outcomes for both ICC and ECC compared to the AJCC system [9,11,15,21,22,23,24,42,46]. Among these studies, LNR values varied between 0.20 and 0.60, with series on distal cholangiocarcinoma (potentially including some ECC) and Asian series frequently presenting lower cutoffs, of around 0.05 to 0.40 [17,47]. In our cohort, a LNR cut-off of 30% was chosen as the most discriminative threshold based on preliminary statistical analysis of our data and a review of commonly used thresholds in the literature.

In cholangiocarcinomas, the efficacy of chemotherapy regimens is not definitively established, and immunotherapies and other targeted therapies are still emerging [48,49,50]. In this cohort, node-positive patients were more likely to receive systemic therapy, with similar rates observed between low and high-risk LNR groups, both of which were higher compared to node-negative disease. Adjuvant strategies were more prevalent than neoadjuvant approaches, which is understandable given the noted combination of nodal disease, higher rates of incomplete resections among these groups, and the tendency to present at advanced stages. The adoption of adjuvant therapy has been largely influenced by the results of the BILCAP trial [50], whereas robust data on neoadjuvant strategies remain scarce [51,52]. Despite ongoing controversies, the administration of any form of systemic therapy was associated with improved survival in our cohort, as was the receipt of radiation therapy. A recent study also leveraging the NCDB database corroborated these findings, demonstrating the benefits of neoadjuvant therapy even in node-negative and margin-negative cholangiocarcinomas [53]. As adjuvant therapies for cholangiocarcinomas progress, the utilization of LNR may play a role in defining which patients would benefit from more aggressive treatments. LNR could assist in guiding the selection of regimens and serve as a useful tool for establishing eligibility criteria for clinical trials in the field.

While LNR has shown promise as a stratification method compared to AJCC nodal staging alone, it is significantly influenced by the total number of lymph nodes harvested [16]. In the setting of suboptimal lymph node retrieval, the log odds of the ratio of disease spread (LODDS) have emerged as a more robust alternative for detailed nodal staging [11]. LODDS represents the natural logarithmic transformation of lymph node ratios and may have an important role in situations where the total number of harvested lymph nodes is not high. In comparison to AJCC and LNR, LODDS has demonstrated a higher accuracy (AUC 0.71 vs. 0.54 and 0.60, respectively) for perihilar cholangiocarcinoma [9]. Despite several studies reporting a somewhat superior performance of LODDS, LNR may be a more practical technique for routine use when a detailed nodal staging evaluation is required. In day-to-day practice, LNR is far easier to calculate than the LODDS for a physician and cutoffs of 30% are easier to integrate in routine than results of logarithmic calculations. Similarly, for risk–benefit discussions, a simple proportion is a more straightforward way for healthcare providers to explain the significance of findings to patients.

This study is subject to several limitations, primarily due to its retrospective nature and reliance on a nationwide database. NCDB lacks individual patient data and specific clinical details that could be relevant as potential confounding variables affecting outcomes. There is also the risk of miscoding and misclassification of procedures and diagnosis, as the accuracy and completeness of the data in the NCDB are reliant on the reporting practices of the participating institutions. Variations in data quality among them may introduce biases or errors. Furthermore, when working with population-level data, the results may not be generalizable to specific subpopulations that may be underrepresented in the database, thereby compromising the external validation of findings.

## 5. Conclusions

LNR is an important prognostic factor for predicting survival outcomes in ICC and ECC patients. It is well-known that LN-negative patients with ICC and ECC have significantly better survival than LN-positive patients. This study strongly demonstrates that survival prognosis can be further stratified based on LNR for ICC and ECC patients and that this is not simply a binary factor.

## Figures and Tables

**Table 1:** Characteristics of the study population by group.

		Primary Site																	
		Intrahepatic Cholangiocarcinoma									Extrahepatic Cholangiocarcinoma								
		LNR 0 (N = 553)		LNR < 30% (N = 223)		LNR ≥ 30% (N = 178)		Total (N = 954)			LNR 0 (N = 680)		LNR < 30% (N = 683)		LNR ≥ 30% (N = 244)		Total (N = 1607)		
		N	%	N	%	N	%	N	%	*p*	N	%	N	%	N	%	N	%	*p*
Age at Diagnosis, years (Median)		66 (58–73)		66 (57–73)		63 (55–70)		65 (57–72)		0.02	68 (60–75)		68 (60–74)		68 (60–75)		68 (60–75)		0.865
Age Group	<65 years	248	44.8%	113	50.7%	99	55.6%	460	48.2%	0.031	278	40.9%	248	36.3%	92	37.7%	618	38.5%	0.215
	≥65 years	305	55.2%	110	49.3%	79	44.4%	494	51.8%		402	59.1%	435	63.7%	152	62.3%	989	61.5%	
Sex	Male	251	45.4%	105	47.1%	92	51.7%	448	47.0%	0.342	444	65.3%	408	59.7%	155	63.5%	1007	62.7%	0.101
	Female	302	54.6%	118	52.9%	86	48.3%	506	53.0%		236	34.7%	275	40.3%	89	36.5%	600	37.3%	
Race	White	463	83.7%	184	82.5%	153	86.0%	800	83.9%	0.072	547	80.4%	540	79.1%	204	83.6%	1291	80.3%	0.425
	Asian	41	7.4%	13	5.8%	16	9.0%	70	7.3%		54	7.9%	47	6.9%	17	7.0%	118	7.3%	
	Black	38	6.9%	17	7.6%	6	3.4%	61	6.4%		62	9.1%	71	10.4%	15	6.1%	148	9.2%	
	American Indian/Aleutian/Eskimo	0	0.0%	1	0.4%	0	0.0%	1	0.1%		1	0.1%	7	1.0%	1	0.4%	9	0.6%	
	Other	10	1.8%	4	1.8%	3	1.7%	17	1.8%		11	1.6%	13	1.9%	4	1.6%	28	1.7%	
	Unknown	1	0.2%	4	1.8%	0	0.0%	5	0.5%		5	0.7%	5	0.7%	3	1.2%	13	0.8%	
Charlson–Deyo Score	0	369	66.7%	166	74.4%	122	68.5%	657	68.9%	0.029	468	68.8%	458	67.1%	162	66.4%	1088	67.7%	0.606
	1	107	19.3%	46	20.6%	36	20.2%	189	19.8%		136	20.0%	134	19.6%	47	19.3%	317	19.7%	
	2	36	6.5%	5	2.2%	12	6.7%	53	5.6%		42	6.2%	47	6.9%	23	9.4%	112	7.0%	
	≥3	41	7.4%	6	2.7%	8	4.5%	55	5.8%		34	5.0%	44	6.4%	12	4.9%	90	5.6%	
Facility Type	Community Cancer Program	13	2.4%	3	1.4%	5	3.0%	21	2.3%	0.108	18	2.7%	23	3.4%	9	3.8%	50	3.2%	0.953
	Comprehensive Community Cancer Program	105	19.4%	57	27.1%	32	19.2%	194	21.1%		152	23.0%	156	23.4%	59	24.6%	367	23.4%	
	Academic/Research Program	318	58.7%	124	59.0%	102	61.1%	544	59.2%		372	56.2%	377	56.5%	132	55.0%	881	56.2%	
	Integrated Network Cancer Program	106	19.6%	26	12.4%	28	16.8%	160	17.4%		120	18.1%	111	16.6%	40	16.7%	271	17.3%	
Distance Traveled, miles (Median)		20.3 (9–66)		17.8 (8–70)		15.3 (7–39)		18.8 (8–61)		0.1	16.8 (3–43)		17.1 (7–49)		20.5 (7–50)		17.6 (7–46)		0.536
NCDB Analytic Stage Group	Stage I	229	41.4%	13	5.8%	1	0.6%	243	25.5%	<0.001	113	16.6%	4	0.6%	0	0.0%	117	7.3%	<0.001
	Stage II	192	34.7%	5	2.2%	5	2.8%	202	21.2%		385	56.6%	295	43.2%	20	8.2%	700	43.6%	
	Stage III	71	12.8%	79	35.4%	83	46.6%	233	24.4%		74	10.9%	309	45.2%	161	66.0%	544	33.9%	
	Stage IV	17	3.1%	91	40.8%	75	42.1%	183	19.2%		7	1.0%	18	2.6%	39	16.0%	64	4.0%	
AJCC Pathologic T	p0	3	1.3%	3	2.9%	0	0.0%	6	1.4%	<0.001	2	1.2%	0	0.0%	1	1.6%	3	0.9%	<0.001
	p1	93	40.4%	8	7.7%	12	15.0%	113	27.3%		22	13.4%	5	4.7%	0	0.0%	27	8.1%	
	p2										2	1.2%	1	0.9%	0	0.0%	3	0.9%	
	p2A	47	20.4%	26	25.0%	15	18.8%	88	21.3%		70	42.7%	24	22.6%	9	14.1%	103	30.8%	
	p2B	26	11.3%	14	13.5%	20	25.0%	60	14.5%		29	17.7%	19	17.9%	21	32.8%	69	20.7%	
	p3	25	10.9%	26	25.0%	15	18.8%	66	15.9%		30	18.3%	55	51.9%	29	45.3%	114	34.1%	
	p4	15	6.5%	10	9.6%	12	15.0%	37	8.9%		5	3.0%	2	1.9%	2	3.1%	9	2.7%	
AJCC Pathologic N	p0	205	89.1%	1	1.0%	1	1.3%	207	49.9%	<0.001	165	99.4%	0	0.0%	1	0.4%	166	49.4%	<0.001
	p1	0	0.0%	87	82.9%	75	93.8%	162	39.0%		0	0.0%	100	94.3%	52	81.3%	152	45.2%	
	p2										0	0.0%	6	5.7%	11	17.2%	17	5.1%	
	NOS/Other	25	10.9%	17	16.2%	4	5.0%	46	11.1%		1	0.6%	0	0.0%	0	0.0%	1	0.3%	
Regional Lymph Nodes Examined, number (Mean)		7.8		12.0		8.1		8.8		<0.001	14.9		19.2		12.5		16.4		<0.001
Regional Lymph Nodes Positive, number (Mean)		0		1.6		4.8		1.3		<0.001	0		2.3		6.1		1.9		<0.001
Surgical Margins	R0	444	80.3%	155	69.5%	101	56.7%	700	73.4%	<0.001	556	81.8%	510	74.7%	144	59.0%	1210	75.3%	<0.001
	R1	65	11.8%	33	14.8%	41	23.0%	139	14.6%		74	10.9%	100	14.6%	58	23.8%	232	14.4%	
	R2	3	0.5%	1	0.4%	2	1.1%	6	0.6%		2	0.3%	1	0.1%	5	2.0%	8	0.5%	
	NOS	41	7.4%	34	15.2%	34	19.1%	109	11.4%		48	7.1%	72	10.5%	37	15.2%	157	9.8%	
Systemic Chemotherapy	No systemic therapy	235	42.5%	50	22.4%	36	20.2%	321	33.6%	<0.001	250	36.8%	186	27.2%	64	26.2%	500	31.1%	<0.001
	Systemic therapy before surgery	84	15.2%	27	12.1%	20	11.2%	131	13.7%		64	9.4%	41	6.0%	7	2.9%	112	7.0%	
	Systemic therapy after surgery	209	37.8%	131	58.7%	104	58.4%	444	46.5%		338	49.7%	439	64.3%	167	68.4%	944	58.7%	
	Systemic therapy both before and after surgery	25	4.5%	15	6.7%	18	10.1%	58	6.1%		28	4.1%	17	2.5%	6	2.5%	51	3.2%	
Radiation	No radiation therapy	454	82.1%	161	72.2%	132	74.2%	747	78.3%	<0.001	503	74.0%	460	67.3%	159	65.2%	1122	69.8%	<0.001
	Radiation therapy before surgery	35	6.3%	7	3.1%	4	2.2%	46	4.8%		43	6.3%	13	1.9%	1	0.4%	57	3.5%	
	Radiation therapy after surgery	61	11.0%	49	22.0%	0	20.2%	146	15.3%		128	18.8%	205	30.0%	79	32.4%	412	25.6%	
	Radiation therapy both before and after surgery	0	0.0%	1	0.4%	1	0.6%	2	0.2%		2	0.3%	0	0.0%	0	0.0%	2	0.1%	

Patient demographics, tumor characteristics, and treatments for intrahepatic (ICC) and extrahepatic (ECC) cholangiocarcinoma, stratified by lymph node ratio (LNR: 0, <30%, ≥30%). R0/R1/R2 denote margin status (no, microscopic, macroscopic residual); NOS = Not Otherwise Specified; IQR = Interquartile Range. *p*-values compare distributions across LNR groups.

**Table 2:** Means and Medians for Survival Times for LNR and LODDS Groups by Primary Site (*p* < 0.001).

	Intrahepatic Cholangiocarcinoma						Extrahepatic Cholangiocarcinoma					
LNR Group	Mean			Median			Mean			Median		
	OS	95% CI		OS	95% CI		OS	95% CI		OS	95% CI	
		Lower	Upper		Lower	Upper		Lower	Upper		Lower	Upper
LNR 0	49.6	47.3	51.9	62.7	-	-	46.2	44.0	48.5	55.7	48.4	63.0
LNR < 30%	39.4	35.6	43.2	40.8	31.5	50.0	33.3	31.0	35.5	27.9	24.4	31.4
LNR ≥ 30%	31.8	27.8	35.8	25.2	21.0	29.4	26.2	23.2	29.2	20.5	16.6	24.5
Overall	44.2	42.4	46.1	48.2	42.4	53.9	38.0	36.5	39.5	35.7	32.8	38.6
LODDS 0	50.2	47.7	52.7	-	-	-	44.7	2.5	46.9	51.6	45.9	57.2
LODDS 1	41.5	38.4	44.7	44.0	3.3	37.5	33.8	31.6	36.0	27.9	24.8	30.9
LODDS 2	31.5	27.5	35.6	25.2	2.3	20.7	25.3	22.1	28.5	20.1	15.8	24.3
Overall	44.2	42.4	46.1	48.2	2.9	42.4	38.0	36.5	39.5	35.7	32.8	38.6

Mean and median overall survival (OS) times for intrahepatic (ICC) and extrahepatic (ECC) cholangiocarcinoma, stratified by lymph node ratio (LNR) and log odds of positive nodes (LODDS). LNR: proportion of positive lymph nodes; LODDS: logarithmic transformation of positive/negative lymph node ratio. Median survival not reached in ICC LODDS0 group. 95% CI = 95% Confidence Interval.

**Table 3:** Cox Regression Analysis by Primary Site.

	Intrahepatic Cholangiocarcinoma								Extrahepatic Cholangiocarcinoma							
	Univariable				Multivariable				Univariable				Multivariable			
	HR	95% CI		*p*	HR	95% CI		*p*	HR	95% CI		*p*	HR	95% CI		*p*
		L	U			L	U			L	U			L	U	
Group: LNR 0	ref			ref	ref			ref	ref			ref	ref			ref
Group: LNR < 3 0%	1.85	1.46	2.34	<0.001	2.08	1.62	2.68	<0.001	1.96	1.66	2.32	<0.001	2.07	1.75	2.46	<0.001
Group: LNR ≥ 30%	2.75	2.16	3.49	<0.001	2.94	2.25	3.84	<0.001	2.89	2.36	3.53	<0.001	2.98	2.41	3.68	<0.001
Age at Diagnosis	1.01	1.00	1.02	0.01	1.01	1.00	1.02	0.01	1.03	1.02	1.03	<0.001	1.02	1.01	1.03	<0.001
Sex	0.81	0.67	0.99	0.04	0.89	0.72	1.08	0.24	0.94	0.81	1.09	0.41	0.91	0.78	1.05	0.19
Charlson–Deyo Score 0	ref			ref	ref			ref	ref			ref	Ref			ref
Charlson–Deyo Score 1	0.98	0.77	1.26	0.90	1.00	0.78	1.29	0.97	1.27	1.06	1.52	0.01	1.26	1.05	1.51	0.01
Charlson–Deyo Score 2	0.77	0.47	1.26	0.30	0.73	0.45	1.21	0.22	1.58	1.23	2.05	<0.001	1.28	0.99	1.66	0.06
Charlson–Deyo Score ≥ 3	0.96	0.62	1.47	0.84	1.08	0.69	1.69	0.74	1.84	1.39	2.44	<0.001	1.60	1.20	2.13	0.00
Histology: ICC 8140	ref			ref	ref			ref	ref			ref	ref			ref
Histology: ICC 8160	0.85	0.61	1.20	0.36	0.92	0.65	1.31	0.66	0.88	0.76	1.02	0.08	0.86	0.75	1.00	0.05
Histology: ICC 8162	0.79	0.11	5.74	0.81	1.08	0.15	8.03	0.94	0.68	0.32	1.43	0.31	0.84	0.40	1.79	0.66
Surgical Margins: R0	ref			ref	ref			ref	ref			ref	ref			ref
Surgical Margins: R1	2.30	1.80	2.95	<0.001	2.02	1.56	2.63	<0.001	1.7	1.4	2.0	<0.001	1.66	1.36	2.02	<0.001
Surgical Margins: R2	3.58	1.48	8.68	0.01	4.56	1.80	11.55	<0.001	1.0	0.4	2.7	0.96	0.87	0.32	2.36	0.79
Surgical Margins NOS	2.06	1.55	2.74	<0.001	1.91	1.43	2.57	<0.001	1.5	1.2	1.9	<0.001	1.43	1.14	1.81	<0.01
Systemic Chemotherapy: None	ref			ref	ref			ref	ref			ref	ref			ref
Systemic Chemotherapy: Neoadjuvant only	0.63	0.45	0.88	0.01	0.63	0.44	0.90	0.01	0.48	0.35	0.66	<0.001	0.82	0.57	1.19	0.3
Systemic Chemotherapy: Adjuvant only	0.76	0.61	0.94	0.01	0.56	0.44	0.71	<0.001	0.59	0.50	0.68	<0.001	0.57	0.48	0.67	<.001
Systemic Chemotherapy: Neoadjuvant + Adjuvant	0.61	0.38	0.97	0.04	0.49	0.30	0.81	<0.001	0.42	0.26	0.67	<0.001	0.49	0.31	0.79	<0.01
Radiation: None	ref			ref	ref			ref	ref			ref	ref			ref
Radiation: Neoadjuvant only	0.68	0.41	1.13	0.14	1.07	0.62	1.85	0.81	0.34	0.20	0.58	<0.001	0.50	0.27	0.91	0.03
Radiation: Adjuvant only	1.01	0.77	1.33	0.93	0.93	0.69	1.25	0.61	0.72	0.61	0.85	<0.001	0.76	0.63	0.92	0.01

Cox regression analysis of overall survival for intrahepatic (ICC) and extrahepatic (ECC) cholangiocarcinoma. LNR: Lymph Node Ratio, HR: Hazard Ratio, 95% CI = 95% Confidence Interval, ref: reference group, NOS: Not Otherwise Specified.

## Data Availability

The data that support the findings of this study (NCDB 2020) are available in the National Cancer Database https://www.facs.org/quality-programs/cancer-programs/national-cancer-database/. Access to these data is not open; permission may be granted upon request to investigators affiliated with Commission on Cancer–accredited facilities.
